# The *GBA* variant E326K is associated with alpha-synuclein aggregation and lipid droplet accumulation in human cell lines

**DOI:** 10.1093/hmg/ddac233

**Published:** 2022-09-20

**Authors:** Laura J Smith, Magdalena M Bolsinger, Kai-Yin Chau, Matthew E Gegg, Anthony H V Schapira

**Affiliations:** Department of Clinical and Movement Neurosciences, UCL Queen Square Institute of Neurology, University College London, Royal Free Campus, London NW3 2PF, UK; Aligning Science Across Parkinson’s (ASAP) Collaborative Research Network, Chevy Chase, MD 20815, USA; Department of Clinical and Movement Neurosciences, UCL Queen Square Institute of Neurology, University College London, Royal Free Campus, London NW3 2PF, UK; Division of Medicine, Friedrich-Alexander University Erlangen-Nurnberg, Schloßplatz 4, 91054 Erlangen, Germany; Department of Clinical and Movement Neurosciences, UCL Queen Square Institute of Neurology, University College London, Royal Free Campus, London NW3 2PF, UK; Aligning Science Across Parkinson’s (ASAP) Collaborative Research Network, Chevy Chase, MD 20815, USA; Department of Clinical and Movement Neurosciences, UCL Queen Square Institute of Neurology, University College London, Royal Free Campus, London NW3 2PF, UK; Aligning Science Across Parkinson’s (ASAP) Collaborative Research Network, Chevy Chase, MD 20815, USA; Department of Clinical and Movement Neurosciences, UCL Queen Square Institute of Neurology, University College London, Royal Free Campus, London NW3 2PF, UK; Aligning Science Across Parkinson’s (ASAP) Collaborative Research Network, Chevy Chase, MD 20815, USA

## Abstract

Sequence variants or mutations in the *GBA* gene are numerically the most important risk factor for Parkinson disease (PD). The *GBA* gene encodes for the lysosomal hydrolase enzyme, glucocerebrosidase (GCase). *GBA* mutations often reduce GCase activity and lead to the impairment of the autophagy-lysosomal pathway, which is important in the turnover of alpha-synuclein, accumulation of which is a key pathological hallmark of PD. Although the E326K variant is one of the most common *GBA* variants associated with PD, there is limited understanding of its biochemical effects. We have characterized homozygous and heterozygous E326K variants in human fibroblasts. We found that E326K variants did not cause a significant loss of GCase protein or activity, endoplasmic reticulum (ER) retention or ER stress, in contrast to the L444P *GBA* mutation. This was confirmed in human dopaminergic SH-SY5Y neuroblastoma cell lines overexpressing GCase with either E326K or L444P protein. Despite no loss of the GCase activity, a significant increase in insoluble alpha-synuclein aggregates in E326K and L444P mutants was observed. Notably, SH-SY5Y overexpressing E326K demonstrated a significant increase in the lipid droplet number under basal conditions, which was exacerbated following treatment with the fatty acid oleic acid. Similarly, a significant increase in lipid droplet formation following lipid loading was observed in heterozygous and homozygous E326K fibroblasts. In conclusion, the work presented here demonstrates that the E326K mutation behaves differently to the common loss of function *GBA* mutations; however, lipid dyshomeostasis and alpha-synuclein pathology are still evident.

## Introduction

Mutations or genetic variants in the *GBA* gene (OMIM 606463) are numerically the most important genetic risk factor for Parkinson disease (PD), increasing the prevalence by 5–30-fold and depending on the mutation, age and ethnicity (1–5). *GBA* mutation-associated PD (*GBA*-PD) leads to an earlier age of onset and increased cognitive impairment (6–8), with alpha-synuclein pathology similar to that of the sporadic PD (6,9). The *GBA* gene encodes the lysosomal hydrolase enzyme, glucocerebrosidase (GCase), which has a primary role of catalyzing the catabolism of the sphingolipids glucosylceramide (GlcCer) and glucosylsphingosine (GlcSph). Additionally, GCase may exert a second catalytic activity, where it is responsible for the transglucosylation of GlcCer to cholesterol to form β-cholesteryl glucoside (GlcChol) (10,11). Homozygous or bi-allelic *GBA* mutations result in the lysosomal storage disorder Gaucher disease.

Mutations in *GBA* are associated with a specific reduction in GCase activity in the brain (12). The degree of pathogenicity associated with each individual *GBA* mutation differs and variants have been stratified into mild (e.g. N370S) or severe variants (e.g. L444P) (13–15). *GBA* polymorphic variants like E326K are referred to as risk variants as they do not cause GD when bi-allelic, yet increase the risk for developing PD in both homozygous and heterozygous forms (16–18). E326K is one of the most prevalent *GBA* variants in PD patients (19–21). In a UK cohort, the prevalence of the E326K variant in PD patients was 7.57%, compared with L444P and N370S that were 1.08% and 2.7%, respectively (22). This variant has been associated with accelerated development of dementia and aggressive motor symptoms (23–25). However, the mechanisms underlying how E326K variants lead to an increased predisposition for PD remain unclear.

The exact mechanisms underpinning the relationship between *GBA* mutations and alpha-synuclein pathology, and how this predisposes some patients to PD, remain elusive. A plethora of models of *GBA*-PD have reported alpha-synuclein pathology (26–31). In cell and animal models of *GBA* mutations, the activation of the unfolded protein response (UPR) (27,32–35) and impairment of the autophagy-lysosome pathway (ALP) (26,27,35–38) have been demonstrated. In a human neural crest stem-cell derived dopaminergic neuron model of *GBA* mutations, a reduction in GCase function was accompanied by increased alpha-synuclein levels and could be rescued with the Gcase small molecule chaperone ambroxol (31). *GBA* mutations have also been linked to altered lipid and cholesterol metabolism in cell and animal models (26,30,35,39–46), with specific species promoting alpha-synuclein aggregation (46–49). Altered lipid profiles have also been reported in the serum of *GBA*-PD patients (50).

The aim of the present study was to first investigate the effect of the E326K variant on the activity and cellular localization of Gcase in fibroblast cells from patients with homozygous and heterozygous E326K mutations. Fibroblasts homozygous for L444P and N370S were utilized as examples of pathogenic *GBA* mutations of the type that can cause GD. The results revealed that the E326K variant is not associated with a significant loss of Gcase activity or protein, unlike L444P and N370S, and does not undergo significant endoplasmic reticulum (ER)-trapping or activate the UPR. These findings were confirmed in SH-SY5Y cells expressing E326K, L444P and N370S Gcase protein. Despite no loss of Gcase activity or activation of the UPR, an accumulation of insoluble alpha-synuclein aggregates was observed in E326K SH-SY5Y cells. A significant elevation in the number of lipid droplets was also demonstrated in fibroblast and SH-SY5Y cells harbouring the E326K variant.

## Results

### No significant reduction in Gcase protein levels in E326K mutant fibroblasts

**Figure 1:**
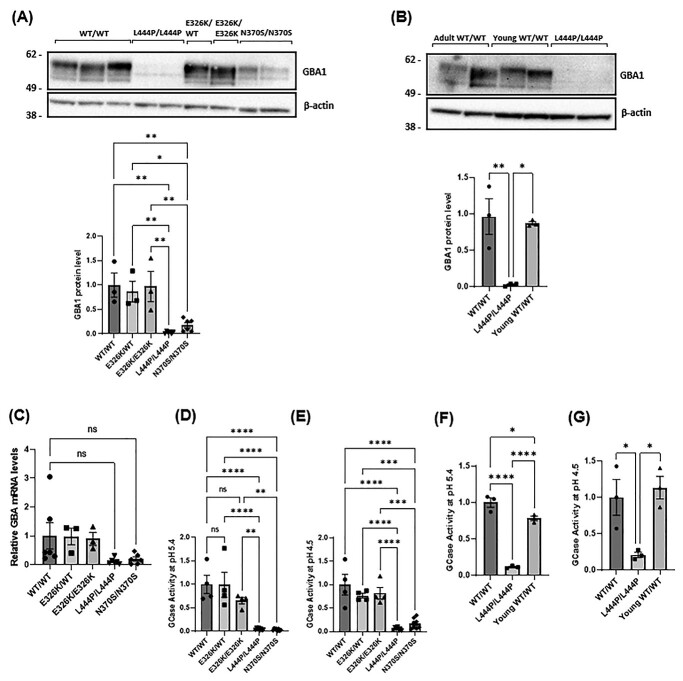
GCase protein level and activity in patient-derived fibroblasts. (**A**) Immunoblot and quantification for the GCase protein level in fibroblasts normalized to WT/WT control fibroblasts as shown graphically as mean with SEM (^*^^*^P < 0.01, ^*^^*^^*^P < 0.001, ^*^^*^^*^^*^P < 0.001; *n* = 3). Three technical repeats. The *n* for each genotype per experiment was WT/WT *n* = 3; E326K/WT *n* = 1; E326K/E326K *n* = 1; L444P/L444P *n* = 2; N370S/N370S *n* = 2. (**B**) Immunoblot and quantification for the GCase protein level in adult and young control fibroblasts normalized to WT/WT control fibroblasts (^*^^*^^*^^*^P < 0.0001; *n* = 3). Three technical repeats. (**C**) The expression of *GBA* mRNA levels in patient fibroblasts was quantified and normalized to WT/WT controls. For each experiment, two biological replicates were used for each cell line. For quantification, *GBA* expression for each cell line was calculated, pooled and averaged for each genotype. Three technical repeats. The *n* for each genotype per experiment was WT/WT *n* = 3; E326K/WT *n* = 1; E326K/E326K *n* = 1; L444P/L444P *n* = 2; N370S/N370S *n* = 2. Graphs show the mean and error bars show the SEM. GCase activity assay with M-Glu was performed at (**D**) pH 5.4 with NaT and (**E**) pH 4.5 on fibroblast cell lysates. All data normalized to WT/WT control fibroblasts. GCase activity in in adult and young control fibroblasts at (**F**) pH 5.4 with NaT and **(G)** pH 4.5. (^*^^*^P < 0.01, ^*^^*^^*^P < 0.001, ^*^^*^^*^^*^P < 0.0001; *n* = 4; ns, not significant). All data normalized to WT/WT control fibroblasts. Three technical repeats. The *n* for each genotype per experiment was WT/WT *n* = 2; young WT/WT *n* = 2; L444P/L444P *n =* 2. All graphs show the mean with error bars as the SEM. Statistical test used was one-away ANOVA with Tukey’s post hoc analysis. Raw data can be found at:https://doi.org/10.5281/zenodo.6985167.

Gcase protein level was assessed in fibroblasts by western blot (Fig. 1A). In this paper, all results were pooled for genotype. Gcase protein and mRNA levels (Fig. 1C) were not significantly different between control and E326K/WT and E326K/E326K lines. In fibroblast lines with homozygous L444P mutations, GCase protein was 2.8% of control (*P* < 0.01), and in fibroblasts with homozygous N370S mutations, GCase protein was 17.5% of control (*P* < 0.01). Because of the young age of the L444P homozygous fibroblast lines, the analysis was repeated with age-matched healthy control fibroblasts. As the homozygous form of L444P generally results in a severe clinical outcome, patients often have a short lifespan, which makes obtaining age-matched cell lines difficult (51). There was no difference in GCase protein levels in young (mean age 9 years) versus older (mean age 61 years) controls (Fig. 1B). L444P/L444P fibroblasts exhibited negligible GCase protein levels compared with both.

### No significant reduction in GCase enzymatic activity in E326K mutant fibroblasts

The total cell GCase activity was measured at pH 5.4 and revealed no significant changes between controls and E326K/WT (99% of control) and E326K/E326K (65.5% of control) (Fig. 1D). A similar pattern was observed at pH 4.5, with E326K/WT cells having 76.6% of control activity and E326K/E326K cells having 82.1% of control activity (Fig. 1E). Both L444P and N370S cells had significantly reduced GCase activity at pH 5.4 (*P* < 0.0001) and pH 4.5 (*P* < 0.0001), compared with controls. Again, results were confirmed in age-matched controls (Fig. 1F–G).

### No significant change in lysosomal content and function in E326K mutant fibroblasts

**Figure 2:**
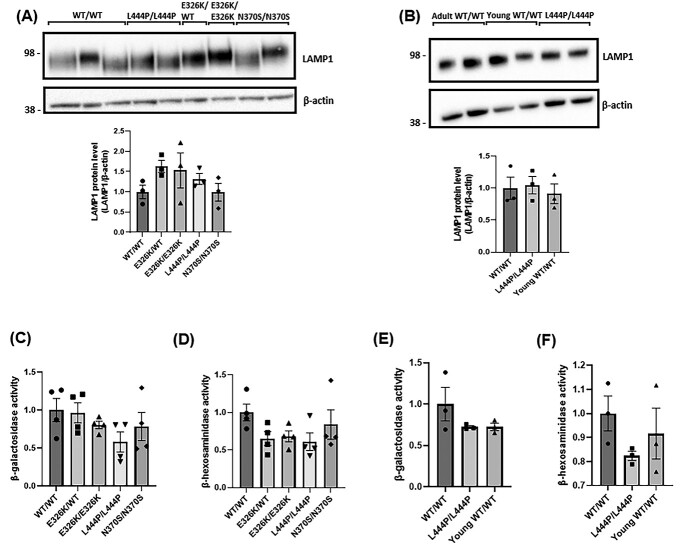
Lysosomal content and function in patient-derived fibroblasts. (**A**) LAMP1 levels were measured via western blot and quantified to assess the overall endo-lysosomal content of the fibroblasts. Data quantified and normalized to WT/WT fibroblast controls. Three technical repeats. (**B**) LAMP1 levels measured via western blot and compared with young control fibroblasts. Lysosomal function in patient-derived fibroblasts. Three technical repeats. (**C**) β-Galactosidase and (**D**) β-hexosaminidase were measured at pH 4.1 to assess the overall lysosomal function in fibroblasts. All data normalized to WT/WT control fibroblasts. Four technical repeats. The activities of lysosomal hydrolases, (**E**) β-galactosidase and (**F**) β-hexosaminidase were measured with young control fibroblasts. Four technical repeats. The *n* for each genotype per experiment was WT/WT *n* = 3; E326K/WT *n* = 1; E326K/E326K *n* = 1; L444P/L444P *n* = 2; N370S/N370S *n* = 2. For the analysis of young controls, WT/WT *n* = 2; young WT/WT *n* = 2; L444P/L444P *n =* 2. All graphs show the mean with error bars as the SEM. The statistical test used was one-away ANOVA with Tukey’s post hoc analysis. Raw data can be found at https://doi.org/10.5281/zenodo.6985167.

The overall endo-lysosomal content of fibroblasts was measured by western blot analysis with the lysosomal marker lysosomal-associated membrane protein 1 (LAMP1) (Fig. 2A). Fig. 2C No significant alterations were observed between the cell lines. No changes were observed in young and old controls (Fig. 2B and D).

To investigate the overall lysosomal function, enzyme activity assays were performed to measure the activity of two other lysosomal hydrolases, β-galactosidase and β-hexosaminidase (Fig. 2C–2D). No significant changes were observed between control lines and mutant fibroblasts. The analysis of age-matched controls revealed no significant alterations between aged and young controls (Fig. 2E-F).

### No significant ER retention of GCase in E326K mutant fibroblasts

**Figure 3:**
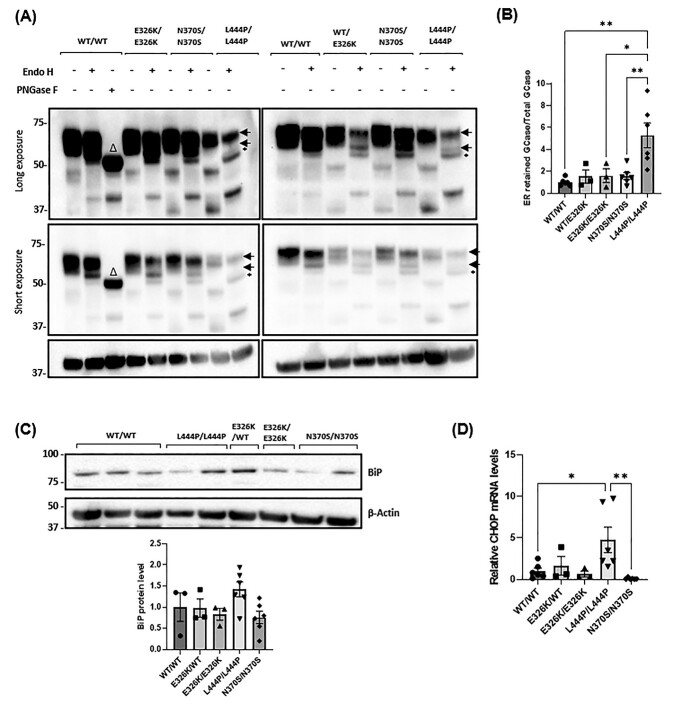
ER retention and ER stress in patient-derived fibroblasts. (**A**) Fibroblast cell lysates (20 μg of WT, E326K and N370S mutants and 70 μg of L444P mutants) were treated with or without endoglycosidase-H (Endo H) and GCase protein species analysed by western blot. WT/WT cell lysate (20 μg) was treated with Peptide-N-Glycosidase F (PNGase F) as a positive control. Figure shows blots at long and short exposures. The two normal species of GCase detected in fibroblasts are indicated by arrows. An additional lower molecular weight band was observed, indicating ER retained GCase, in L444P/L444P fibroblasts following endo-H treatment (black asterisk). The WT/WT cell line treated with PNGase exhibited a lower molecular weight band, indicating a GCase species with no glycosylation (Δ). (**B**) Quantitative analysis of Endo H digestion displayed as the density of bands for ER resident GCase divided by the density of bands for total GCase protein, normalized to the β-actin band density. Data normalized to WT/WT that was set at 1. Three technical repeats. The *n* for each genotype per experiment was WT/WT *n* = 2; E326K/WT *n* = 1; E326K/E326K *n* = 1; L444P/L444P *n* = 2; N370S/N370S *n* = 2. ER stress was analysed by (**C**) quantifying the BiP protein level in fibroblasts. Results are normalized to WT/WT control fibroblasts. Three technical repeats. (**D**) The expression of *CHOP* mRNA levels in patient fibroblasts was quantified and normalized to WT/WT controls. For each experiment, two biological replicates were used for each cell line. For quantification, *CHOP* expression for each cell line was calculated, pooled and averaged for each genotype. Three technical repeats. The *n* for each genotype per experiment was WT/WT *n* = 3; E326K/WT *n* = 1; E326K/E326K *n* = 1; L444P/L444P *n* = 2; N370S/N370S *n* = 2. Graphs show the mean with error bars showing the SEM. The statistical test used was one-way ANOVA with Tukey post hoc analysis (^*^P < 0.05, ^*^^*^P < 0.01; *n* = 3). Raw data can be found at https://doi.org/10.5281/zenodo.6985167.

The different glycosylation patterns of GCase, as it passes through the secretory pathway, can be utilized to assess the proportion of GCase that is trapped in the ER. ER-retained forms of GCase carry N-linked glycans that are sensitive to cleavage by Endo-H, producing lower molecular bands on western blot. The GCase protein resolves as three bands after treatment, with the lower exposure blot showing two higher molecular weight bands likely to be mature protein (Fig. 3A). For the analysis, wild-type fibroblasts were digested by PNGase F as a positive control for unglycosylated Gcase (Δ symbol on the blot). The corresponding ER-resident bands in the mutant cell lines are marked with a black asterisk. This band is absent in wild-type cell lines. To quantify the retention of mutant GCase protein, the density of the ER-resident band was divided by the density of the two mature GCase bands (Fig. 3B). Compared with controls, L444P/L444P fibroblasts were the only cells to exhibit an increase in the Endo-H sensitive GCase fraction (5.28-fold) (*P* < 0.01).

To further investigate GCase trafficking, lysosomal GCase activity was measured using a substate that is taken up only in to acidic vesicles and fluoresces upon catalysis with GCase (52). Lysosomal GCase activity was measured in E326K/+, E326K/E326K and L444P/L444P fibroblasts (Supplementary Material, Fig. S1). After loading of the substrate, there is a linear increase in fluorescent product up to 45 min, after which the GCase enzymatic activity plateaus. This plateau is the depletion/release of substrate from late endosomes and lysosomes over time. Therefore, the initial linear rate of the enzyme activity was calculated between time 0 and 45 min. Lysosomal GCase activity was abolished in wild-type fibroblast cells pretreated with 10 M conduritol-B-epoxide (CBE) for 24 h, a GCase-specific inhibitor and similar to lysosomal GCase activity in L444P/L444P fibroblasts. In comparison, E326K/WT and E326K/E326K fibroblasts have activity in the lysosome closer to that of controls, suggesting this variant behaves differently to the L444P loss of the function variant.

### No significant ER stress in E326K mutant fibroblasts

To assess UPR activation in fibroblasts, BiP chaperone protein levels were measured (Fig. 3C). BiP (GRP78) is a central regulator of the UPR, binding to unfolded proteins in the ER and activating the three arms of the UPR. Expression levels can also be increased during ER stress (53). No significant changes were observed in any mutant lines compared with healthy controls.

Analysis of *CHOP* mRNA expression, which is upregulated in response to prolonged activation of the UPR following the binding of BiP to the PERK arm of the UPR (54), revealed a 4.75-fold increase in L444P/L444P fibroblasts (*P* < 0.05) (Fig. 3D). No significant increases were observed in E326K or N370S fibroblasts compared with controls.

### Characterization of the GCase protein level, expression and activity in SH-SY5Y cell lines expressing GCase mutants

**Figure 4:**
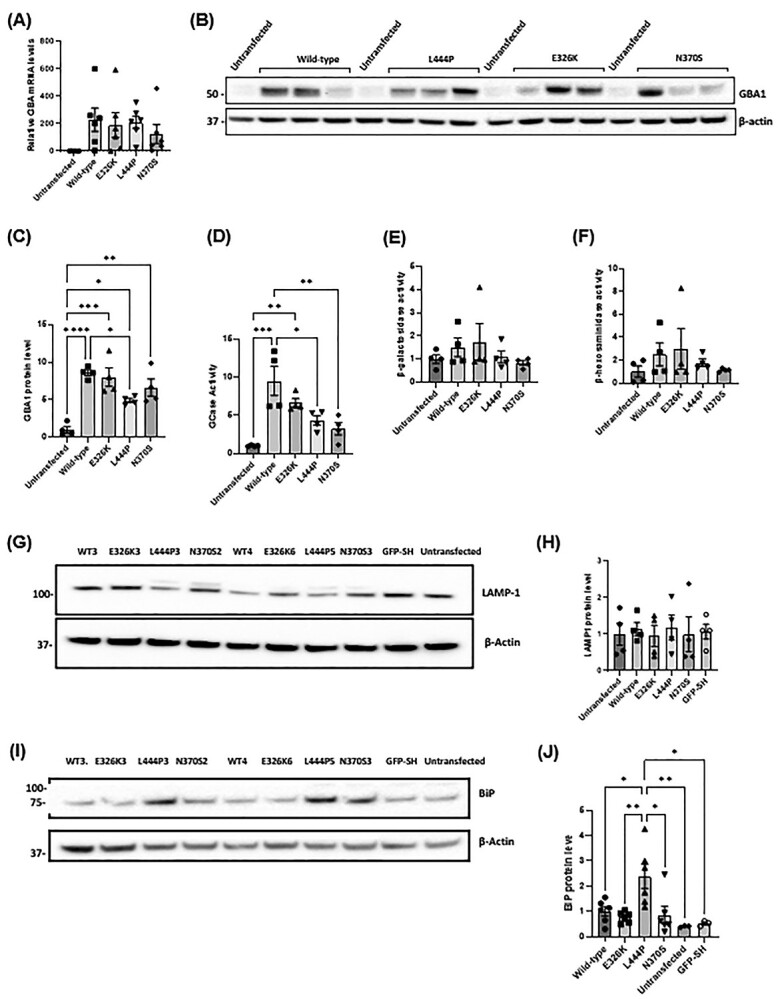
*GBA* levels, lysosomal function and ER stress in SH-SY5Y cells overexpressing mutant GCase. (**A**) Quantification of *GBA* mRNA levels in stable SH-SY5Y cell lines, normalized to untransfected SH-SY5Y cells (set at 1). Three technical repeats. The *n* for each genotype per experiment was wild type *n* = 2; E326K *n* = 2; L444P *n* = 2; N370S *n* = 2; untransfected *n* = 1. (**B**) Immunoblot and (**C**) quantification of *GBA* protein levels in stable SH-SY5Y cell lines. Data normalized to untransfected SH-SY5Y cells (set at 1). Graphs show the mean with SEM. The statistical test used was one-way ANOVA with Tukey’s post hoc analysis (^*^P < 0.05, ^*^^*^P < 0.01, ^*^^*^^*^P < 0.001, ^*^^*^^*^^*^P < 0.0001; *n* = 4). Four technical repeats. (**D**) GCase activity in nmole/hr/mg in stable SH-SY5Y cell lines measured at pH 5.4 with NaT and normalized to untransfected SH-SY5Y cells (set at 1) Graphs show the mean with SEM. The statistical test used was one-way ANOVA with Tukey’s post hoc analysis (^*^P < 0.05, ^*^^*^P < 0.01, ^*^^*^^*^P < 0.001; *n* = 4). The activities of lysosomal hydrolases, β-galactosidase and β-hexosaminidase were measured at pH 5.1 in SH-SY5Y stable cell lines. Four technical repeats. (**E**) β-galactosidase activity in nmole/0.5 h/mg in undifferentiated SH-SY5Y clones normalized to untransfected SH-SY5Y cells. (**F**) β-Hexosaminidase activity in nmole/0.5 h/mg measured in undifferentiated SH-SY5Y clones normalized to untransfected SH-SY5Y cells. Four technical repeats. (**G**) LAMP1 levels were measured via western blot and (**H**) quantified to assess the overall endo-lysosomal content of the SH-SY5Y cell lines. Immunoblot and quantification for LAMP1 protein level in SH-SY5Y stable cell lines. Data normalized to wild-type SH-SY5Y cells. Four technical repeats. (**I**) Immunoblot and (**J**) quantification of BiP protein level in SH-SY5Y clones. Data normalized to untransfected SH-SY5Y cells. Six technical repeats. The *n* for each genotype per experiment was wild type *n* = 2; E326K *n* = 2; L444P *n* = 2; N370S *n* = 2; untransfected *n* = 1; GFP *n* = 1. Graphs show the mean with SEM. The statistical test used was one-way ANOVA with Tukey’s post hoc analysis(^*^P < 0.05. ^*^^*^P < 0.01; *n* = 3). Raw data can be found at https://doi.org/10.5281/zenodo.6985167.

SH-SY5Y stable cell lines expressing wild-type, E326K, L444P and N370S *GBA* constructs were generated and data from two clones pooled for each genotype. Overexpression of *GBA* was confirmed via qPCR (Fig. 4A). Wild-type (288-fold), E326K (190-fold), L444P (209-fold) and N370S (125-fold) lines all expressed similar higher levels of GCase mRNA when compared with untransfected cells.

Compared with untransfected SH-SY5Y cells, all GCase overexpressing lines had higher GCase protein levels and activity (*P* < 0.05; 0.0001) (Fig. 4B–D). No significant changes in the GCase protein level or activity were observed between wild-type and E326K cells. Cells expressing L444P mutant protein had lower GCase protein levels (55.4% of wild type) (*P* < 0.05) and activity (45.5% of wild type) (*P* < 0.05) compared with wild type. In N370S cells, the GCase protein level was not significantly reduced unlike activity, but it was 33.6% of wild type (*P* < 0.01).

### Overall lysosomal content and function unaltered by GCase mutations in undifferentiated SH-SY5Y cells

No significant changes were observed in activity of the lysosomal hydrolases, β-galactosidase and β-hexosaminidase, and protein level of LAMP1 across SH-SY5Y cell lines (Fig. 4E–H). These data indicate that *GBA* mutations do not influence the overall endolysosomal content in SH-SY5Y cells.

### Increased ER stress in undifferentiated SH-SY5Y cells overexpressing L444P mutant GCase

Activation of the UPR in SH-SY5Y cells was investigated by measuring BiP protein levels. Compared with wild type, cells expressing L444P GCase protein were the only *GBA* lines that exhibited a significant elevation in BiP levels (*P* < 0.05) (Fig. 4I–J). This was also significantly higher than E326K (*P* < 0.01) and N370S (*P* < 0.05) lines. As a control, cells overexpressing green fluorescent protein, which has a similar size to GCase, also did not increase BiP levels. Note that *CHOP* mRNA levels in SH-SY5Y were extremely low (>30 cycles) and could not be measured reliably.

### Increased soluble intracellular alpha-synuclein protein level in undifferentiated SH-SY5Y cells overexpressing L444P mutant GCase

**Figure 5:**
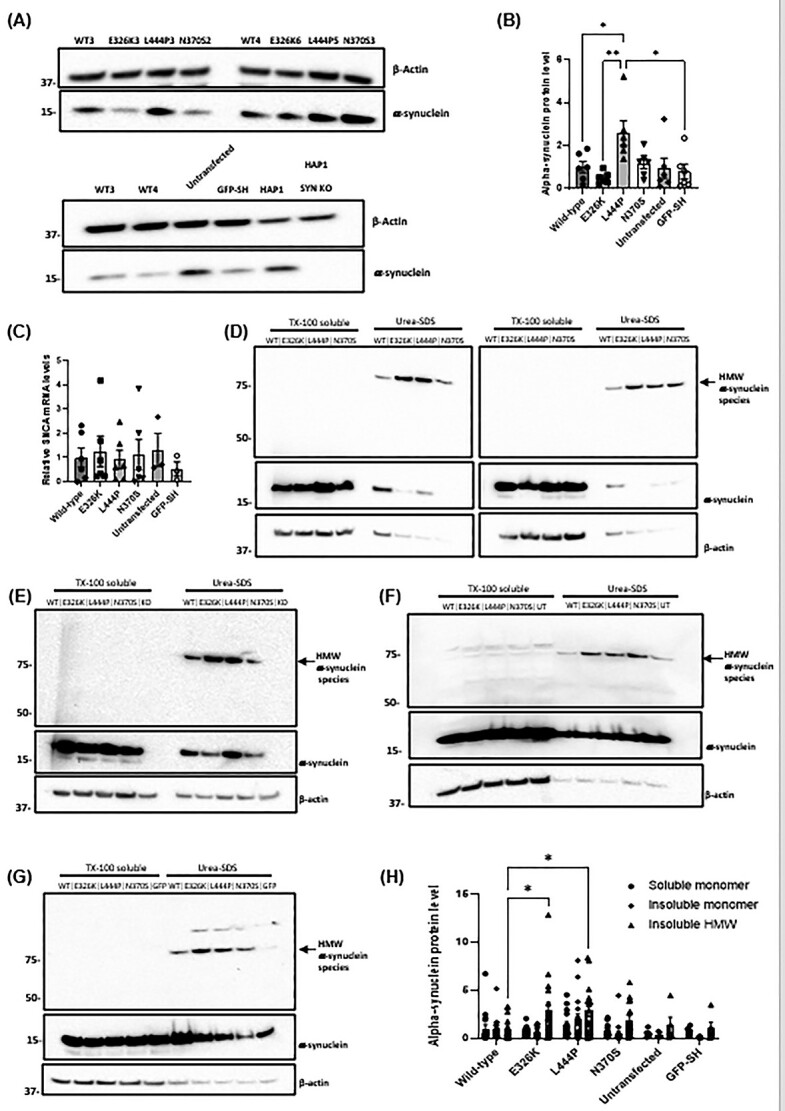
Soluble and insoluble alpha-synuclein levels in SH-SY5Y stable cell lines expressing mutant GBA. (**A**) Immunoblot of alpha-synuclein protein level in SH-SY5Y clones. (**B**) Quantification of alpha-synuclein immunoblotting in SH-SY5Y clones normalized to wild-type clones. The *n* for each genotype per experiment was wild type *n* = 2; E326K *n* = 2; L444P *n* = 2; N370S *n* = 2; untransfected *n* = 1; GFP *n* = 1. Six technical repeats. (**C**) Quantification of *SNCA* mRNA levels in SH-SY5Y stable clones normalized to wild-type clones (*n* = 3). The *n* for each genotype per experiment was wild type *n* = 2; E326K *n* = 2; L444P *n* = 2; N370S *n* = 2; untransfected *n* = 1; GFP *n* = 1. Two biological repeats and six technical repeats. TX-100 soluble and insoluble fractions (urea-SDS) were made from cells and analysed for alpha-synuclein by western blotting. HMW alpha-synuclein species (arrow) were detected in urea-SDS fractions. Monomeric alpha-synuclein was present in the TX-100 soluble fraction and some urea-SDS fractions. (**D**) Immunoblot for all SH-SY5Y cell lines over-expressing mutant *GBA* protein. Appropriate control cell lines were also analysed: (**E**) HAP1 alpha-synuclein knockout cells (KO), (**F**) untransfected SH-SY5Y cells (UT) and (**G**) GFP over-expressing SH-SY5Y cells (GFP). (**H**) Quantification of soluble and insoluble alpha-synuclein immunoblots. Data normalized to wild-type clones. The *n* for each genotype per experiment was wild type *n* = 2; E326K *n* = 2; L444P *n* = 2; N370S *n* = 2; untransfected *n* = 1; GFP *n* = 1. Ten technical repeats. Graphs show the mean with SEM. The statistical test used was one-way ANOVA with Tukey’s post hoc analysis or two-way ANOVA with Tukey’s post hoc analysis (^*^P < 0.05; *n* = 16). Raw data can be found at https://doi.org/10.5281/zenodo.6985167.

Accumulation of alpha-synuclein monomers in cells is a key feature in models of *GBA*-PD (26,29,37,38,43,52,55). Analysis of Triton X-100 soluble, monomeric alpha-synuclein revealed no significant changes in E326K and N370S cells compared with wild type (Fig. 5A–B), whereas those expressing the L444P variant exhibited a 2.58-fold increase (*P* < 0.05). There were no significant changes in *SNCA* mRNA expression observed between cell lines (Fig. 5C).

### Increased insoluble alpha-synuclein protein level in undifferentiated SH-SY5Y cells overexpressing mutant GCase protein

Under pathological conditions, alpha-synuclein can form insoluble, phosphorylated aggregates (56). The accumulation of these aggregates is a main pathological feature of PD, and thus alpha-synuclein aggregates are a fundamental protein in the study and modelling of the disease (57). The effect of *GBA* mutations on the accumulation of alpha-synuclein aggregates in the Triton X-100 insoluble fraction was investigated in SH-SY5Y cells. Soluble and insoluble Triton X-100 fractions were run on the same western blot and immunoblotted for alpha-synuclein (Fig. 5D–H). The vast majority of alpha-synuclein remained in the soluble fraction. Monomeric, soluble alpha-synuclein was a single band at the expected molecular weight of 15 kDa. No significant changes in soluble alpha-synuclein monomers were observed.

There was evidence of an high molecular weight (HMW) alpha-synuclein species in the insoluble fraction (75 kDa), which was absent in human *SNCA* knockout HAP1 lines (Fig. 5E). Insoluble alpha-synuclein band density was expressed as a ratio against soluble β-actin (Fig. 5H). As expected in the insoluble fraction, β-actin band density was lower than the soluble fraction and likely represents β-actin associated with Triton X insoluble membranes (e.g. lipid rafts). There was a concurrent and significant increase in E326K (2.88-fold) and L444P (2.91-fold) cells compared with wild type (*P* < 0.05), indicative of an accumulation of insoluble alpha-synuclein aggregates.

### Increased number of lipid droplets in E326K mutant cell lines

**Figure 6:**
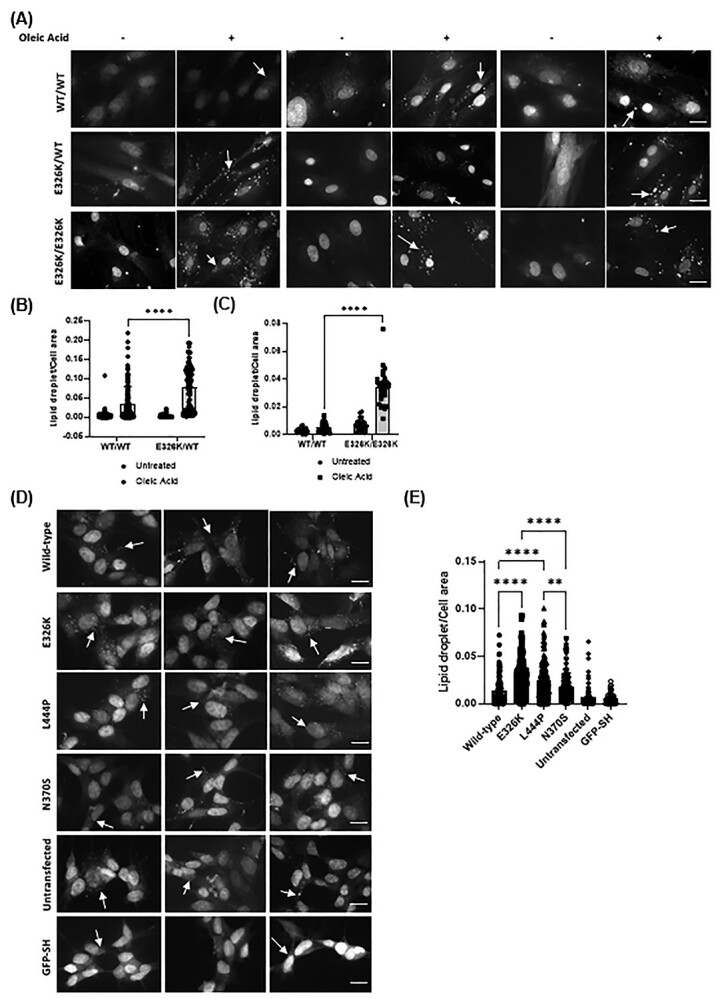

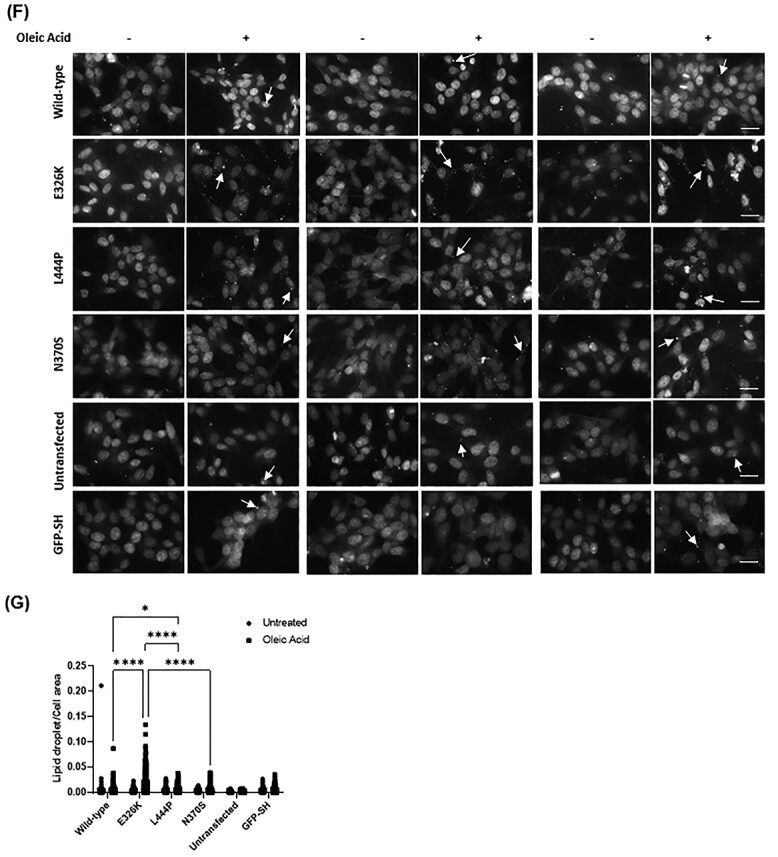
Lipid droplets in GBA mutant cells. (**A**) Fibroblast lines harbouring the E326K mutations were starved in Opti-MEM overnight and incubated with and without 100 μM OA for 5 h. Cells were stained with lipophilic fluorescent probe BODIPY 493/503. Lipid droplets represent punctate structures. Three representative images from each genotype shown (−) denotes untreated and (+) denotes treated with OA. All cells were counted in the images and the number of lipid droplets counted and normalized to cell area using ImageJ. Scale bar represents 20 μm. The *n* for each genotype per experiment was WT/WT *n* = 6; E326K/WT *n* = 1; E326K/E326K *n* = 1; >100 cells analysed per genotype. Quantification of lipid droplets in (**B**) control and E326K/+ fibroblasts and (**C)** control and E326K/E326K fibroblasts displayed as the mean with error bars showing SEM. The statistical test used was one-way ANOVA with Tukey post hoc analysis or two-way ANOVA with Tukey’s post hoc analysis (^*^^*^^*^^*^P < 0.0001). (**D**) SH-SY5Y cells over-expressing mutant GCase protein were grown on coverslips and stained with lipophilic fluorescent probe BODIPY 493/503. Lipid droplets represent punctate structures. Three representative images from each genotype shown. All cells were counted in the images and the number of lipid droplets counted and normalized to cell area using ImageJ. The *n* for each genotype per experiment was wild type *n* = 2; E326K *n* = 2; L444P *n* = 2; N370S *n* = 2; untransfected *n* = 1; GFP *n* = 1. > 50 cells analysed per genotype. (**E**) Quantification of lipid droplets per cell area shown as the mean with SEM. The statistical test used was one-way ANOVA with Tukey post hoc analysis or two-way ANOVA with Tukey’s post hoc analysis (^*^^*^P < 0.01; ^*^^*^^*^^*^P < 0.0001). (**F**) SH-SY5Y cells over-expressing mutant GCase protein were starved in Opti-MEM overnight and incubated with and without 100 μM OA for 5 h. Cells were stained with lipophilic fluorescent probe BODIPY 493/503. Lipid droplets represent punctate structures. Three representative images from each genotype shown (−) denotes untreated and (+) denotes treated with OA. All cells were counted in the images and the number of lipid droplets counted and normalized to cell area using ImageJ. The *n* for each genotype per experiment was wild type *n* = 2; E326K *n* = 2; L444P *n* = 2; N370S *n* = 2; untransfected *n* = 1; GFP *n* = 1. > 50 cells analysed per genotype. (**G**) Quantification of lipid droplets per cell area displayed as mean with error bars showing SEM. Statistical test used was one-way ANOVA with Tukey post hoc analysis or two-way ANOVA with Tukey’s post hoc analysis (^*^P < 0.05; ^*^^*^^*^^*^P < 0.0001). Scale bar represents 20 μm. Raw data can be found at https://doi.org/10.5281/zenodo.6985167.

As *GBA* mutations have been associated with altered lipid metabolism (58–60), lipid droplet accumulation was measured in our cell lines. Lipid droplets are organelles involved in intracellular lipid homeostasis (61). BODIPY 493/503 stains neutral lipids and lipid droplets are visualized as punctate structures, examples of which are shown by arrows (Fig. 6). The number of lipid droplets were quantified using Image J and normalized to cell area. To further analyse the effect of *GBA* mutations on lipid droplet accumulation, SH-SY5Y cells were grown under basal conditions and stained with BODIPY 493/503 (Fig. 6D–E). In these conditions, a higher number of lipid droplets was observed in mutants compared with wild type, significantly elevated in E326K (2.1-fold) (*P* < 0.0001; *n* = 3) and L444P (1.8-fold) (*P* < 0.0001). Both were significantly higher compared with N370S expressing lines (E326K *P* < 0.0001 and L444P *P* < 0.01). The number of lipid droplets in untransfected cells was significantly lower than all over-expressing cell lines (wild type *P* < 0.05; E326K, L444P and N370S *P* < 0.0001). However, SH-SY5Y cells overexpressing GFP were not significantly different from untransfected cells, suggesting this increase in lipid droplet number is not simply an artifact of protein overexpression.

To investigate if the increased LD number in *GBA* mutant cells was because of increased rate of lipid droplet formation, SH-SY5Y cells were starved overnight in OptiMEM and then treated with the fatty acid oleic acid (OA) for 5 h to induce lipid droplet formation (62) (Fig. 6F–G). A 5.1-fold increase in lipid droplet formation was observed in E326K cells compared with wild-type cells (*P* < 0.0001). In SH-SY5Y cell lines expressing L444P GCase protein, a 1.77-fold increase in lipid droplets, compared with wild type, was observed (*P* < 0.05). Lipid droplet formation in E326K cells was significantly higher than in L444P (*P* < 0.0001), N370S (*P* < 0.0001) and GFP (*P* < 0.0001) lines. Both E326K and L444P were significantly higher than untransfected (E326K *P* < 0.0001 and L444P *P* < 0.001).

To confirm the role of E326K in LD formation, fibroblast lines heterozygous and homozygous for E326K were starved overnight, loaded with OA and lipid droplet formation quantified (Fig. 6A–C). Compared with control cells, E326K/WT cells exhibited a 2.11-fold increase in lipid droplet formation following lipid loading with OA (*P* < 0.0001) (Fig. 6B). Similarly, following OA treatment, a 6.61-fold increase in lipid droplet formation was observed in E326K/E326K cells compared with controls (Fig. 6C).

## Discussion

**Figure 7:**
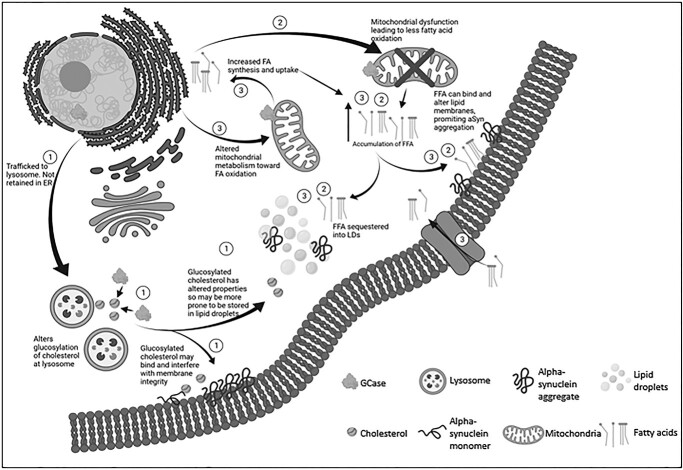
Proposed mechanisms underlying E326K GCase pathology. (1) E326K GCase is likely not retained in the ER and is trafficked to the lysosome. At the lysosome, GCase carries out its ‘moonlighting’ function of glucosylating cholesterol. E326K Gcase may have increased or decreased the ability to glucosylate cholesterol, which alters the properties of accumulated cholesterol. This cholesterol can interfere with lipid membranes, altering lipid composition and promoting alpha-synuclein aggregation. This cholesterol has altered properties so may be more prone to storage in to lipid droplets, leading to an accumulation of lipid droplets. Lipid droplets can act as a site to bind alpha-synuclein, and in high concentrations and under pathological conditions, alpha-synuclein may aggregate at lipid droplets. (2) The E326K GCase mutation may lead to dysfunctional mitochondria, potentially through impaired clearance. These dysfunctional mitochondria may have reduced ability to metabolize fatty acids, leading to the accumulation of FFA in the neuron. FFA are capable of interfering with lipid membranes, potentially promoting alpha-synuclein aggregation. FFA are also sequestered into lipid droplets, as the neuron attempts to protect the cell from lipotoxicity. (3) The E326K mutation may cause a shift in the mitochondria’s metabolism capacity, shifting away from glycolysis toward fatty acid oxidation to provide energy for the neuron. In order to meet the demand for fatty acids, the neuron may be more primed to synthesize fatty acids or take up external fatty acids. This may lead to an accumulation of FFA in the neuron, which can exert lipotoxic effects, accumulate in lipid droplets and may induce alpha-synuclein aggregation.

The results of this study indicate that the E326K variant does not behave in a similar fashion to the pathogenic *GBA* mutations L444P and N370S. Notably, despite no significant loss of E326K GCase activity, insoluble alpha-synuclein aggregates in SH-SY5Y cells were observed and coincident with an enhanced formation and accumulation of lipid droplets in SH-SY5Y and fibroblast lines, suggestive of altered lipid homeostasis. A schema highlighting the potential pathways underlying the mechanism of the E326K variant is shown in Figure 7.

Few studies have biochemically characterized E326K GCase protein. This study provides evidence that E326K in fibroblasts does not induce a significant loss of Gcase function or result in an UPR, unlike other common pathogenic *GBA*-PD mutations N370S and L444P. In homozygous and heterozygous forms, E326K mutations are not associated with a significant loss of GCase expression or activity in fibroblasts and SH-SY5Y cells. These findings are supported by previous literature demonstrating the E326K variant generally reduces GCase activity to a lesser extent than other pathogenic *GBA* mutations (18,63–68), whereas L444P and N370S variants are reported to induce a loss of GCase function (33–35,47,68–73), as demonstrated in this study. However, it is important to note that the E326K variant may behave differently depending on the species or cell type, as in two previous studies in COS-1 and HeLa cells, there is a reduction in GCase activity of around 75% (33,74).

Aberrant trafficking and retention of mutant GCase in the ER has been reported in a variety of cell models (33,34,68,69,75–78). Activation of the UPR has been demonstrated extensively in cells harbouring the L444P variant (32,34,68,75,77,78). In this study, unlike L444P, the E326K protein is not localized to the ER in fibroblasts and does not activate the UPR, suggesting correct lysosomal trafficking and the absence of a severely misfolded protein. It may be that the mutant E326K protein exerts a gain of function mechanism through a ‘moonlighting’ function such as the glucosylation of cholesterol at the lysosome (10). The altered properties of glucosylated cholesterol may increase its storage in lipid droplets or influence the lipid membrane fluidity, which may promote the aggregation of alpha-synuclein (79). Alterations to the composition of lipid rafts, which are central regulators of chaperone mediated autophagy (CMA) (80), may impair CMA-mediated degradation of alpha-synuclein. Furthermore, an increase in cholesterol has been associated with an impairment of autophagy in N370S fibroblasts (35). As ER retention of GCase is reported to correlate with disease severity (81) and has been shown to be variable among lines harbouring the same N370S genotype (77), this may explain the lack of impaired GCase localization in N370S cells in this study.

Evidence suggests a role for *GBA* mutants in the accumulation of alpha-synuclein monomers and aggregates (82). Despite no loss of GCase activity, SH-SY5Y cells expressing the E326K variant exhibit an increase in insoluble alpha-synuclein aggregates. This was increased to a similar level as L444P mutant cells. As the L444P variant has been shown to accelerate alpha-synuclein pathology and spread in mice (83,84), this points toward a potentially similar propagation of pathology in E326K carriers. The observation that the E326K mutation occurs on the GCase protein surface (85) suggests protein interactions may be influenced. It is possible that the E326K mutant GCase protein has a reduced affinity for binding alpha-synuclein, as suggested in the N370S protein (86), which could induce alpha-synuclein lipid binding and aggregation.

Accumulation of insoluble alpha-synuclein aggregates may be explained by the accumulation of lipid droplets in E326K fibroblasts and SH-SY5Y cells, suggestive of an altered lipid metabolism. Lipid homeostasis is necessary for maintaining proper function of the neuron and synaptic plasticity. Alterations in such pathways have been reported in PD with increased levels of TAGs, cholesterol and ceramides (87). In an effort to overcome the lipid overload, cells initially induce a pathway to store excess lipids in lipid droplets (61). Although suggested to be initially protective (88,89), excess lipid droplets can be neurotoxic (90). Lipid droplets protect cells from excess fatty acids that can be targeted for lipid peroxidation. During oxidative stress, reactive oxygen species can attack free fatty acids (FFA) and generate toxic lipid peroxides and reactive aldehydes, which can exert lipotoxicity, including damage to lipid membranes, ER stress, mitochondrial damage and subsequent neurodegeneration (88,89,91–94). Excessive lipid droplet formation has however been demonstrated to trigger neurotoxicity (90). It may be this that contributes to neurodegeneration in E326K mutants.

Under normal conditions, the number of lipid droplets accumulating in SH-SY5Y cells expressing E326K and L444P was elevated compared with wild-type cells. Following lipid loading, lipid droplet formation was significantly elevated in these cells compared with wild-type and other mutant lines. The same was observed in heterozygous and homozygous E326K fibroblasts. Interestingly, although L444P SH-SY5Y cell lines exhibited increase lipid droplets after lipid loading, this was to a much lesser extent than E326K lines. As the E326K variant is not associated with a loss of GCase function or activation of the UPR, yet the L444P line is, it is likely that these variants induce accumulation of lipid droplets through different pathways. The L444P variant may lead to improper ALP and clearance of lipids, whereas the E326K variant may work through pathways involving the mitochondria and metabolism of lipids.

Many studies demonstrate a correlation between increased lipid droplets and alpha-synuclein pathology (89,95,96). Recently, in a mouse model of synucleinopathy, lipid droplet accumulation correlated with alpha-synuclein pathology, both of which were rescued by overexpressing wild-type Gcase (97), reinforcing the role of Gcase in this pathway. Proper regulation of cellular lipids is critical to maintain the composition and fluidity of lipid membranes. Such lipids can bind alpha-synuclein and accelerate its formation into toxic oligomeric and fibrillar species, propagating PD pathology. In addition, alterations in lipid membrane integrity can influence alpha-synuclein binding and enhance the conversation of monomeric alpha-synuclein into toxic aggregates (87). The altered composition of GSLs has been demonstrated in L444P and N370S neurons levels (26,27,49), although the accumulation of these lipids in *GBA*-PD is debatable (98,99). A shift toward short-chain GSLs may be induced by *GBA* mutations, which can promote the toxic aggregation of alpha-synuclein (47).

The E326K mutation may influence lipid metabolism, possibly inducing its remodelling toward fatty acid oxidation, priming neurons to more efficiently synthesize or take up fatty acids. Fatty acid oxidation occurs in the mitochondria (100) and a defective mitochondrial network may contribute to E326K pathogenesis (101). Mitochondrial dysfunction has long been implicated in the pathogenesis of PD and associated with *GBA* mutations (36,102–104). There may also be a reciprocal relationship between mitochondria and lipid droplets as they may associate and form mitochondria with a unique structure and function less prone to fatty acid oxidation (101).

Understanding the effect of the E326K variant in human midbrain dopamine neurons is an exciting avenue to explore in future work, including the potential role of mitochondria in the mechanism of disease. This may shed light on why the E326K variant contributes to the risk of developing PD, but does not lead to GD.

In conclusion, this study supports the hypothesis that the mechanisms associated with individual *GBA* mutations may be multiple and provides evidence that the E326K variant does not behave in the same way as the common loss of function variants, L444P and N370S.

## Materials and Methods

### Cell lines

The majority of human fibroblast cell lines were taken from the Schapira laboratory cell line bank. Participants gave informed consent and the collection of skin biopsies was approved by the Royal Free Research Ethics Committee (REC number 10/H0720/21) and the Great Ormond Street Hospital Ethics Committee. Cell lines GM10905 (Coriell Cat# GM20272, RRID:CVCL_0R42) and GM20272 (Coriell Cat# GM20272, RRID:CVCL_0R42) (L444P/L444P) were purchased from the Coriell Institute cell repository. ND41016 (Subject ID: NDS00203) (NHCDR Cat# ND41016, RRID:CVCL_EZ71) (E326K/E326K) was purchased from NINDS Stem Cell Catalogue. Cell lines UCL-CTRL001 (WT/WT) (RRID:CVCL_B7T4); UCL-CTRL002 (WT/WT) (RRID:CVCL_B7T5); UCL-CTRL003 (WT/WT) (RRID:CVCL_B7T6); UCL-YCTRL001 (WT/WT) (RRID:CVCL_B7T8); UCL-E001K (WT/E326K) (RRID:CVCL_B7T9); UCL-N001S (N370S/ N370S) (RRID:CVCL_B7TA) and UCL-N002S (N370S/N370S) (RRID: CVCL_B7TB) were generated in the Schapira laboratory and are being deposited to ATCC and will be available by final publication. Fibroblast cell line 7301 was obtained from the MRC Centre for Neuromuscular Diseases Biobank London, supported by the National Institute for Health Research Biomedical Research Centres at Great Ormond Street Hospital for Children NHS Foundation Trust and at University College London Hospitals NHS Foundation Trust and University College London. Details of the fibroblast cell lines used in this study can be found in Supplementary Material, Table S1. Parental SH-SY5Y cells were purchased from ATCC (ATCC Cat# CRL-2266, RRID:CVCL_0019).

### Fibroblast cell culture

Fibroblasts were cultured in Dulbecco’s modified eagle media 4500 (mg/l) growth medium supplemented with Glutamax (Invitrogen), 10% fetal bovine serum, non-essential amino acids (0.1 mM of glycine, L-alanine, L-asparagine, L-aspartic acid, L-glutamic acid, L-proline and L-serine) and penicillin/streptomycin antibiotic cocktail (50 ng/ml) at 37°C and 5% CO_2_ (dx.doi.org/10.17504/protocols.io.rm7vzy542lx1/v1). Analyses were carried out at low passages, and disease and control cultures were matched for the passage number.

### Generation of SH-SY5Y cell lines

Proliferating SH-SY5Y neuroblastoma cell lines were cultured as previously described (37) (dx.doi.org/10.17504/protocols.io.bp2l617jzvqe/v1). SH-SY5Y cells were transfected with a pcDNA™3.1^(+)^ mammalian expression vector (ThermoFisher, Cat#V79020) containing wild type (WT GBA pcDNA3.1; Addgene plasmid #188580; RRID: Addgene_188 580), E326K (E326K GBA pcDNA3.1; Addgene plasmid #188581; RRID: Addgene_188 581), L444P (L444P GBA pcDNA3.1; Addgene plasmid #188582; RRID: Addgene_188 582) or N370S (N370S GBA pcDNA3.1; Addgene plasmid #188583; RRID: Addgene_188 583), *GBA* cDNA. Mutations were introduced by site directed mutagenesis, according to the manufacturer’s guidelines (Agilent Technologies QuikChange II Site-Directed Mutagenesis Kit) (https://www.agilent.com/cs/library/usermanuals/public/200523.pdf). Stable transfection was performed using the XtremeGENE reagent for 72 h, and 400 μg/ml G418 antibiotics are used as the selection marker (dx.doi.org/10.17504/protocols.io.rm7vzy54rlx1/v1). Colonies were selected and expanded for a routine culture in growth media supplemented with G418. For the analysis, two clones per genotype were used.

### SDS-PAGE and western blotting

Cells were lysed in 1% (v/v) Triton X-100 in phosphate-buffered saline (PBS) lysis buffer with protease and phosphatase inhibitors. Cell lysates containing 10–30 μg of protein were electrophoresed with NuPage™ 4–12% Bis-Tris protein gels. Proteins were transferred to a polyvinylidene fluoride (PVDF) membrane, blocked in 10% milk and treated with primary and secondary antibodies in 5% milk. For the analysis of alpha-synuclein, an additional step was added prior to blocking to fix the membrane with 4% paraformaldehyde (PFA) (w/v) and 0.01% (v/v) glutaraldehyde for 30 min. Antibody binding was detected using the GE Healthcare Amersham™ electro-chemi-luminescence™ Prime Western Blotting Detection Reagent (dx.doi.org/10.17504/protocols.io.261genwyyg47/v1). The following antibodies were used: GBA clone 2e2 (Millipore Cat# AP1140, RRID:AB_10683318, dilution 1:1000); GRP78/BiP (Abcam Cat# ab21685, RRID:AB_2119834, dilution 1:1000); monomeric alpha-synuclein (Abcam Cat# ab138501, RRID:AB_2537217, dilution 1:1000); LAMP1 (Novus Cat# NB120–19294, RRID:AB_788858, dilution 1:1000); β-actin (Abcam Cat# ab8227, RRID:AB_2305186, dilution 1:10 000).

### Triton X-100 extraction of soluble and insoluble alpha-synuclein

SH-SY5Y cells were trypsinized and lysed in 1% (v/v) Triton X-100, 50 mM Tris, pH 7.5, 750 mM NaCl, 5 mM EDTA, 4 units RQ1 RNase-free DNase (Promega), protease and phosphatase inhibitor mix (Halt) on ice for 15 min. The lysate was passed through a 23-G needle and pelleted at 17 000 g for 30 min at 4°C. Triton X 100 soluble fraction was collected and protein concentration measured using the BCA (Bicinchoninic Acid) Protein Assay.. Insoluble pellets were resuspended in 2% (w/v) sodium dodecyl sulfate (SDS), 8 M urea, 10 mM Tris, pH 7.5, 4 units RQ1 RNase-free DNase, protease and phosphatase inhibitor mix and incubated for 15 min at room temperature. Debris was removed by centrifugation at 17 000 × g for 30 min at 4°C (dx.doi.org/10.17504/protocols.io.6qpvr6p1ovmk/v1).

### Quantitative real-time PCR of mRNA

RNA was isolated from cells using the QIAGEN RNeasy mini kit (dx.doi.org/10.17504/protocols.io.ssbeean). Quantitative analysis of mRNA was performed as previous (105) (dx.doi.org/10.17504/protocols.io.q26g74xoqgwz/v1). GAPDH was amplified as the reference mRNA. Relative gene expression was calculated using the ΔC_T_ method. All results obtained were from the evaluation of two technical duplicates of three independent experiments.

### Total cellular lysosomal enzyme assays

Cells were lysed in 1% (v/v) Triton X-100 in PBS with protease and phosphatase inhibitors. Activity was measured as described previously (12). GCase activity was measured in McIIvaine buffer at pH 5.4 in the presence of 22 mM sodium taurocholate (NaT) and at pH 4.5, with 5 mM methylumbelliferyl-β-D-glucopyranoside (M-Glu) substrate (dx.doi.org/10.17504/protocols.io.n2bvj625nlk5/v1). β-Galactosidase and β-hexosaminidase activity was measured in the McIIvaine buffer at pH 4.1 with 1 mM 4-methylumbelliferyl β-D-galactopyranoside and 2 mM 4-methylumbelliferyl *N*-acetyl-β-D-glucosaminide substrates, respectively (dx.doi.org/10.17504/protocols.io.kqdg3p8r7l25/v1).

### Real-time lysosomal GCase assay

Lysosomal GCase activity, but not ER and Golgi-resident GCase activity, can be measured in live cells by using 5-(pentafluorobenzoylamino) fluorescein Di-β-d-glucopyranoside (PFB-FDGlu; ThermoFisher) as a substrate that is taken up into acidic vesicles only, where it fluoresces upon catalysis with GCase (52) (dx.doi.org/10.17504/protocols.io.eq2lyn8mqvx9/v1). Fibroblasts were grown to 70–90% confluency and the real-time GCase activity assay was performed as described previously (105). The CBE-sensitive initial rate was calculated and normalized to protein concentration in the well. Cells were measured in triplicates.

### Endo H analysis

Fibroblasts were lysed in 1% (v/v) Triton X-100 in PBS and protein concentration determined by a BCA assay. Digestions were performed according to the manufacturer’s instructions (New England BioLabs #P0702L and #P0704S) (dx.doi.org/10.17504/protocols.io.8epv59ew4g1b/v1 and (dx.doi.org/10.17504/protocols.io.cqfvtm). For wild-type E326K and N370S mutant lines, 20 μg of total protein was denatured and for L444P mutants 60 μg of the total protein was denatured. Following this, the denatured protein sample was incubated for 2 h at 37°C, with 500 000 units/ml of either Endo H or PNGase F and analysed by western blotting.

### Staining for lipid droplets

Cells were grown on coverslips until 50–80% confluent. Analysis was performed on SH-SY5Y cells cultured in normal media as well as fibroblasts and SH-SY5Y cells treated for 5 h with 100 μM OA following over-night starvation in Opti-MEM (ThermoFisher). OA is a potent inducer of lipid droplet formation (62). Cells were treated with 250 μl BODIPY™ 493/503 (4,4-Difluoro-1,3,5,7,8-Pentamethyl-4-Bora-3a,4a-Diaza-*s*-Indacene) diluted in PBS at a final concentration of 1 mg/ml for 15 min in the dark at room temperature. Following incubation, coverslips were washed three times in PBS and mounted onto glass slides with 1 μg/ml DAPI CitiFluor for the analysis on a Zeiss Axioplan microscope (QImaging wLS). Images were captured using Micro-Manager software (RRID:SCR_000415; http://micro-manager.org) and analysed using ImageJ software version 1.51j8 (RRID:SCR_003070; https://imagej.net/) with a macro to calculate the number of lipid droplets and cell area (dx.doi.org/10.17504/protocols.io.q26g74xqqgwz/v1).

### Statistics

Graphs were made using GraphPad Prism version 9.3.1 (RRID:SCR_ 002798; http://www.graphpad.com/) and data expressed as the mean ± standard error of the mean (SEM). Statistical significance was determined by one-way analysis of variance (ANOVA) and Tukey’s post hoc or two-way ANOVA and Tukey’s post hoc test using GraphPad Prism version 9.3.1. Data distribution was assumed as normal without formal testing.

## Acknowledgements

The MRC Centre for Neuromuscular Diseases Biobank London is acknowledged for providing the fibroblast cell line 7301.

*Conflict of Interest statement.* None declared.

## Open access

For the purpose of open access, the author has applied a CC BY 4.0 public copyright license to all Author Accepted Manuscripts arising from this submission.

## Funding

Parkinson’s UK (grant number: G-1704); Kattan Trust; Aligning Science Across Parkinson’s (grant number: ASAP-000420) through the Michael J. Fox Foundation for Parkinson’s Research (MJFF); National Institute for Health Research University College London Hospitals Biomedical Research Centre (to A.H.V.S.); Erasmus+ Program (Friedrich-Alexander University, Erlangen, Germany to M.M.B.); National Institute for Health Research Biomedical Research Centres at Great Ormond Street Hospital for Children NHS Foundation Trust and at University College London Hospitals NHS Foundation Trust and University College London.

## Supplementary Material

Supplementary_Figures_Smith_2022_ddac233

Supplementary_Tables_Smith_2022_ddac233
