# Supplementary material for: The GBA variant E326K is associated with alpha-synuclein aggregation and lipid droplet accumulation in human cell lines
Source: Hum Mol Genet. 2022 Sep 20;32(5):773–89. doi: 10.1093/hmg/ddac233 (PMC9941838; doi:10.1093/hmg/ddac233)
Supplement: Supplementary_Figures_Smith_2022_ddac233 [file supplementary_figures_smith_2022_ddac233.pdf]

Legends to Figures

(A)

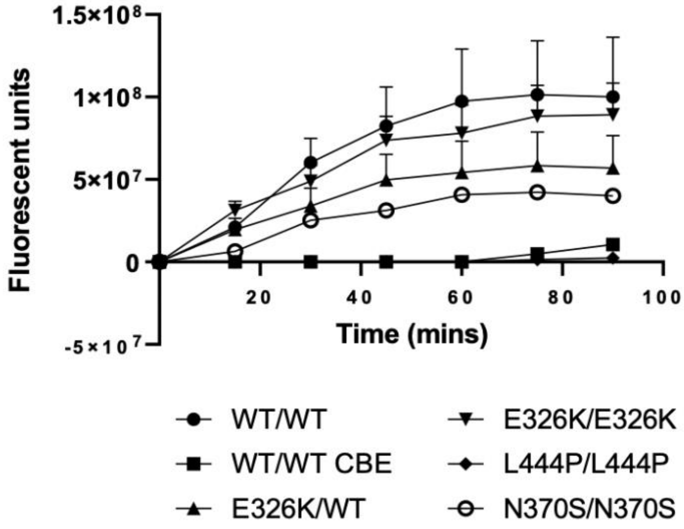

(B)

Genotype	Linear trendline equation (y=mx)
WT/WT	y=18403371x
WT/E326K	y=11332758x
E326K/E326K	y=16823338x
L444P/L444P	y=0x
N370S/N370S	y=7128687x
WT/WT + CBE	y=0x

Supplementary Figure 1. Activity of lysosomal GCase in patient fibroblast lines. (A) Fibroblast cell lines were incubated with PFB-FDGluc substrate for 1 hour at 37°C. Following washing, fluorescence was measured every 15 minutes for 90 minutes. **(B)** The initial linear rate of each reaction was calculated between time 0 and time 45 minutes and initial rate equations displayed in the table. The *n* for each genotype per experiment was WT/WT *n*=3; E326K/WT *n*=1; E326K/E326K *n*=1; L444P/L444P *n*=2; N370S/N370S *n*=1. Statistical test used was one way ANOVA with Tukey post-hoc analysis. Activity at 45 minutes: WT/WT vs. L444P/L444P ***p<0.001; WT/WT vs. WT/WT + CBE **p<0.01; E326K/E326K vs. L444P/L444P *p<0.05. Raw data can be found at: https://doi.org/10.5281/zenodo.6985167
