# Supplementary material for: The GBA variant E326K is associated with alpha-synuclein aggregation and lipid droplet accumulation in human cell lines
Source: Hum Mol Genet. 2022 Sep 20;32(5):773–89. doi: 10.1093/hmg/ddac233 (PMC9941838; doi:10.1093/hmg/ddac233)
Supplement: Supplementary_Tables_Smith_2022_ddac233 [file supplementary_tables_smith_2022_ddac233.pdf]

Legends to Tables

Supplementary Table 1. Characteristics of fibroblast cell lines. For fibroblast analysis cell lines used were: UCL-CTRL001, UCL-CTRL002, UCL-CTRL003, 7301, UCL-YCTRL001, UCL-E001K, ND41016, GM10905, GM20272, UCL-N001S and UCL-N002S.

Cell line	GBA genotype	Phenotype	Gender	Age
UCL-CTRL001	WT/WT	Unaffected	F	58
UCL-CTRL002	WT/WT	Unaffected	M	53
UCL-CTRL003	WT/WT	Unaffected	F	73
7301	WT/WT	Unaffected	F	14
UCL-YCTRL001	WT/WT	Unaffected	M	1
UCL-E001K	WT/E326K	PD	M	50
ND41016	E326K/E326K	PD	M	52
GM10905	L444P/L444P	Type I GD	M	7
GM20272	L444P/L444P	Type II GD	M	Child
UCL-N001S	N370S/N370S	GD	F	58
UCL-N002S	N370S/N370S	GD	M	76
